# Complete case logistic regression with a dichotomised continuous outcome led to biased estimates

**DOI:** 10.1016/j.jclinepi.2022.11.022

**Published:** 2023-02

**Authors:** Rosaleen Peggy Cornish, Jonathan William Bartlett, John Macleod, Kate Tilling

**Affiliations:** a Population Health Sciences, Bristol Medical School, University of Bristol, Oakfield House, Oakfield Grove, Bristol BS8 2BN, UK; b MRC Integrative Epidemiology Unit, University of Bristol, Bristol, UK; c Department of Medical Statistics, London School of Hygiene & Tropical Medicine, WC1E 7HT, London, UK; d NIHR ARC West, 9th Floor, Whitefriars, Lewins Mead, Bristol, BS1 2NT, UK

**Keywords:** Logistic regression, Complete case analysis, Missing data, Multiple imputation, ALSPAC, Auxiliary variable

## Abstract

**Objectives:**

To investigate whether a complete case logistic regression gives a biased estimate of the exposure odds ratio (OR) if missingness depends on a continuous outcome, but a binary version is used for analysis; to examine whether any bias could be reduced by including a misclassified form of the incomplete outcome as an auxiliary variable in multiple imputation (MI).

**Study Design and Setting:**

Analytical investigation, simulation study, and data from a UK cohort.

**Results:**

There was bias in the exposure OR when the probability of being a complete case was independently associated with the exposure and (continuous) outcome but this was generally small unless the association with the outcome was strong. Where exposure and (continuous) outcome interacted in their effect on this probability, the bias was large, particularly at high levels of missing data. Inclusion of the auxiliary variable resulted in important bias reductions when this had high sensitivity and specificity.

**Conclusion:**

The robustness of logistic regression to missing data is not maintained when the outcome is a binary version of an underlying continuous measure, but the bias will be small unless the association between the continuous outcome and missingness is strong.

**What is new?**

**Key findings**
• A complete case logistic regression will give a biased estimate of the exposure odds ratio if the probability of being a complete case depends on a continuous outcome but a binary version of this outcome is used in the analysis; this bias is likely to be small unless the association between the continuous outcome and the chance of being a complete case is strong. If there is an interaction between the exposure and outcome in terms of the probability of being a complete case, there could be substantial bias in the estimate of the log odds ratio.
• If an interaction is present, including one or more auxiliary variables that are good predictors of the missing binary outcome in multiple imputation (MI), models will lead to relatively large bias reductions if these variables have high sensitivity and specificity in relation to the binary outcome; if not, the bias reductions will be small.

**What this adds to what was known?**
• It is known that a complete case logistic regression will give an unbiased estimate of the exposure odds ratio if the probability of being a complete case depends on the outcome and exposure independently. We show that this does not hold when the probability of being a complete case depends on an underlying continuous outcome and a binary form of this is used for analysis.

**What is the implication and what should change now?**
• If one or more good predictors of the missing outcome are available, we would recommend using MI over a complete case analysis because, in practice, it would be difficult to rule out an interaction.

## 1 Introduction

Epidemiological studies often suffer from missing data arising through non-response. This results in a loss of power and may induce bias. One of the most common approaches to addressing missing data is to carry out a complete case analysis, in which the analysis is restricted to individuals with complete data on all variables in the analysis model.

For continuous outcomes analyzed using linear regression, a complete case analysis will give an unbiased estimate of the exposure-outcome association unless the probability of being a complete case depends on the outcome after taking account of the exposure and confounders [1]. For binary outcomes analyzed using logistic regression, the estimate of the (exposure) odds ratio (OR) will be unbiased unless the log probability of being a complete case depends linearly on the exposure and the outcome and–additionally–an interaction between the exposure and outcome [2].

However, although many outcomes in medicine are binary (e.g., diagnosed with type II diabetes), they are often based on one or more underlying continuous measures. For example, a person is diagnosed with type II diabetes if their blood glucose exceeds a defined threshold. Many diseases are not simply present or absent and, as such, the binary measure is a crude version of an underlying continuous outcome. For example, although tools exist to determine whether an individual meets a diagnostic threshold for depression, it is widely acknowledged that mental disorders such as depression are best measured on a continuum [3]. Although such outcomes are often treated as binary variables in epidemiological studies, it is likely that missingness–if related to the outcome–would depend on levels of the underlying continuous measure (e.g., symptom severity) rather than being only dependent on whether an individual meets the diagnostic threshold. If the underlying continuous outcome were analyzed using linear regression, this association of missingness with the outcome would cause the exposure-outcome coefficient to be biased in a complete case analysis. If missingness were directly associated with a binary form of the outcome, this would not cause any bias in the estimated OR for exposure and outcome. In the current study, we aim to examine whether the robustness of logistic regression to missingness related to the outcome still holds if missingness is associated with an underlying continuous outcome (and the outcome in the analysis is a dichotomized version of this continuous outcome).

We firstly derive an expression for the complete case log OR (i.e., the OR targeted by a complete case analysis) and specifically examine the situation where the probability of being a complete case depends independently on the exposure and the underlying continuous outcome. We use this expression to show which factors determine the magnitude of the bias in the complete case OR. We then investigate this in practice using data from the Avon Longitudinal Study of Parents and Children (ALSPAC), examining the association between smoking in pregnancy (exposure) and offspring depression (dichotomised outcome). We also use measures of depression derived from linked general practitioner (GP) data to explore the likely missing data mechanism and as auxiliary variables in multiple imputation (MI). We compare the estimate obtained from MI to that obtained in the complete case analysis. Finally, we present results from a simulation study, based on this example, exploring in which situations using a misclassified form of the missing outcome as an auxiliary variable in MI reduces (or increases) bias compared to a complete case analysis. Here, we vary the sensitivity of the auxiliary variable (linked GP measure of depression) in relation to the binary outcome.

## 2 Methods

The general expression for the full population exposure OR and the complete case OR are derived in the supplementary material. These were used to calculate the bias in the complete case OR, assuming that the continuous outcome was normally distributed (conditional on the binary exposure), that the binary outcome depended on the continuous outcome via a logistic function, and that missingness depended on the continuous outcome via a logistic function. Full details are given in the supplementary materials and Supplementary Figure S1 shows the assumed relationship between the continuous and the binary outcome.

ALSPAC provided the motivating example for the current study. ALSPAC is a birth cohort which recruited 14,500 pregnant women living in and around Bristol, a city in the south west of England, in the early 1990s. Detailed data were collected during pregnancy and the offspring have been followed up since birth. Further details are given elsewhere [4,5]. ALSPAC has a searchable data dictionary and variable search tool [6].

### 2.1 Linkage to general practitioner data

In ALSPAC, informed parental consent was mandatory until age 16. When the children reached legal adulthood, they were sent ‘fair processing’ materials describing ALSPAC's intended use of their health and administrative records and were given means to consent or object. Data were not extracted for participants who objected, or who were not sent fair processing materials. Linkage to GP data is described in the supplementary material.

### 2.2 Analysis of ALSPAC data

The outcome was a binary measure of depression (meets ICD-10 criteria for a diagnosis, yes/no, derived from the revised Clinical Interview Schedule (CIS-R) [7], completed during a study clinic attended when participants were 18 years. Note that the CIS-R also can be used to generate a (numerical) depression score, but this was not used in our ALSPAC analysis. The exposure was maternal smoking in pregnancy (yes/no); this was based on the questionnaire data collected during pregnancy and shortly after birth. We adjusted for the following confounders measured during pregnancy: maternal age, parity and educational level, maternal and paternal antenatal anxiety and depression, family occupational social class, housing tenure (home owned/mortgaged, privately rented, rented from the local council, or a housing association), and number of rooms in the home (excluding bathrooms). We also adjusted for sex because of its strong association with the outcome.

From the linked GP data we derived three binary measures of depression: whether or not an individual had a (1) current, (2) historical, or (3) future record of a diagnosis, symptoms or treatment for depression (henceforth referred to as current, historical, or future depression). The time periods these outcomes relate to are as follows. Current refers to the period 6 months either side of the month in which the CIS-R was completed, historical refers to any time prior to this period, and future any time following this period up until the 31st December 2016 (the last date for which data were extracted). These measures and their association with the binary depression indicator defined using the CIS-R, have been described previously [8].

We used generalized linear models for a binary outcome to examine associations with the probability that ALSPAC-measured depression was observed (outcome = 1 if ALSPAC-measured depression was observed; 0 if missing). We used a logit link (logistic regression) to estimate OR describing these relationships and a log link (log-linear model) to estimate risk ratios (RR). For a binary outcome, if the probability of being observed given the exposure and outcome cannot be factorized as a product of (a function of) the exposure and (a function of) the outcome, this implies a multiplicative interaction in a binomial regression model with a log link; however, binomial regression with a log link can often fail to converge (so estimates cannot be obtained) because the log link does not constrain probabilities to be less than 1. Logistic regression–using a complete case analysis and MI using chained equations–was used to examine the association between smoking in pregnancy and offspring depression. In addition to all the variables included in the substantive model (smoking in pregnancy, binary CIS-R depression status, and covariates described above), the MI models included the following auxiliary variables: the three measures of depression derived from GP data and whether the mother had ever smoked (collected via questionnaire in early pregnancy but referring to lifetime smoking). Stata's *mi impute chained* command was used to carry out the imputations; 100 datasets were imputed with a burn-in of 20 iterations.

### 2.3 Simulation study

#### 2.3.1 Simulated datasets

We first simulated complete datasets of 10,000 observations (to approximately match the numbers in ALSPAC with complete baseline covariates). Missing data were then simulated in a separate process. The variables simulated were analogous to: depression (binary outcome), a numerical depression score, maternal smoking in pregnancy (exposure), and current GP-recorded depression (auxiliary variable). For simplicity, we did not simulate additional covariates. The variables were simulated such that their distributions–and relationships between them–were similar to those observed in ALSPAC. Technical details and formulae for the processes described below are given in the supplementary material. In summary, maternal smoking in pregnancy was simulated to give a prevalence of 25%. The continuous depression score was simulated as a standard normal variable (following a normal distribution with a mean of 0 and variance of 1) conditional on maternal smoking in pregnancy. The binary depression measure was created using the depression score to give a prevalence of 15%.

The analysis model was a logistic regression with the binary outcome (depression) and the exposure (maternal smoking in pregnancy, also binary). Thus, the estimate of interest is the log OR for depression comparing those whose mother smoked during pregnancy to those whose mother did not smoke. The data were generated such that this log OR was 0.405 (giving an OR of 1.50).

For the purposes of this analysis, the study measure of depression was taken as the reference standard. Thus, the linked (binary) GP measure of depression was created to give different sensitivities (25% and 75%) and a specificity of 97.5% in relation to the study's binary measure.

#### 2.3.2 Generating the missing data

We created missing data only in the outcome (both the continuous and binary version); this was simulated as missing not at random (MNAR) in two ways:

(i) The probability of the outcome being observed was only associated with the continuous outcome
(ii) The log probability of the outcome being observed depended linearly on the exposure, continuous outcome, and their interaction (note that, henceforth, where we refer to an interaction, this is what we mean)

In both sets of scenarios, different percentages of missing data were generated. Note that in the latter set of scenarios a complete case analysis is not expected to be unbiased.

#### 2.3.3 Scenarios investigated

The following four factors were varied in the simulations:

Factor 1: Percent missing outcome data.

Factor 2: Sensitivity of GP depression measure in determining study binary depression.

Factor 3: Interaction (yes/no) between exposure and continuous outcome with respect to probability of being observed.

Factor 4: Percent missingness in linked GP depression measure.

The scenarios are summarized in Table 1. In the scenarios without missingness in the linked GP depression measure, we simulated every possible combination of Factors 1-3; in the set of scenarios where 25% missingness was introduced in the linked variable (Factor 4), only Factor 1 was varied; the other factors were fixed: interaction present, sensitivity of GP measure 75%. For each scenario we simulated 1,000 datasets.

**Table 1:** Scenarios investigated in the simulations (each investigated at all four levels of Factor 1: 20%, 40%, 60%, and 80% missing outcome data)

Factor 4: % Missing linked data	Factor 3: Interaction between outcome & exposure with respect to probability of being observed	Factor 2: Sensitivity of GP depression
0%	No	25%
0%	No	75%
0%	Yes	25%
0%	Yes	75%
25%	Yes	75%

#### 2.3.4 Statistical analysis

We estimated the log OR for depression on smoking in pregnancy using logistic regression. We used a complete case analysis and MI–in which the missing (binary) study measure of depression was imputed using logistic regression from the exposure and the GP measure of depression. For each simulated dataset, 100 datasets were imputed.

The estimates obtained from these analyses were compared to the full-population log OR. The bias was estimated as $\bar{\ln OR}-full\;population\;\ln OR$, where $\bar{\ln OR}$ was the estimated log OR averaged over the 1,000 simulated datasets. This was converted to percentage bias. We also calculated the empirical standard error, the standard deviation of the point estimates for the log OR. For MI, we also calculated the fraction of missing information – a measure that quantifies the loss of information due to missing data in multiply imputed data [9] – and the percent increase in precision compared to the complete case analysis, given by the standard error of the log OR obtained using a complete case analysis divided by the standard error obtained from MI.

The simulations and all data analysis were carried out in Stata; R was used to numerically evaluate the integrals in the expression derived for the complete case odds ratio.

## 3 Results

### 3.1 Bias in complete case analysis

**Fig. 1:**
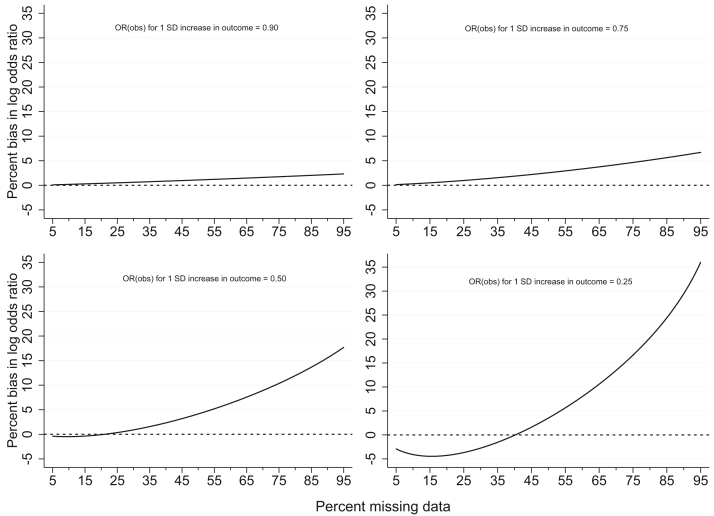
Percent bias in log OR from a complete case analysis for a full population exposure log OR of 0.405 and when the probability of being observed only depends on the (continuous) outcome: varying percentage of missing data and strength of association between continuous outcome and missingness.

The expression derived in the supplementary material indicates that, when the probability of being observed depends independently on the exposure and continuous outcome, the complete case odds ratio (the odds ratio consistently estimated by a complete case analysis) does not depend on the marginal distribution of the exposure (i.e., in this case the prevalence of maternal smoking in pregnancy) or on the relationship between the exposure and missingness (see Supplementary Material). Figure 1 shows how the bias in the complete case log odds ratio varies as the percentage of missing data and the strength of association between the continuous outcome and the probability of being observed changes where the full population log odds ratio is equal to 0.405 (OR = 1.50) and for the specified relationship between the continuous and binary outcome (Equation 7 and Fig. S1 in Supplementary Material). When the association between the continuous outcome and the probability of being observed is weak (OR = 0.90) the complete case bias is very small, even at very high levels of missing data. The bias increases as the association between the outcome and the chance of being observed becomes stronger and as the amount of missing data increases. Supplementary Figure S2 shows the percent bias when the relationship between the outcome and the chance of being observed is reversed, such that the probability of being observed increases rather than decreases as the outcome increases. In this situation, there is negative bias in the log odds ratio (for a full population log odds ratio of 0.405).

### 3.2 Simulation study

**Fig. 2:**
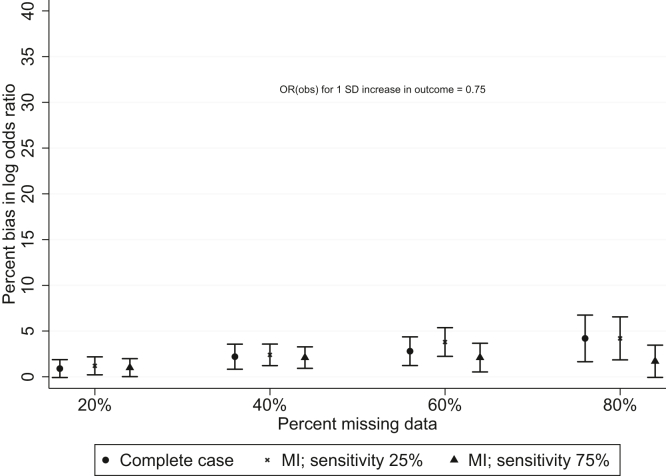
Mean percent bias in log OR when the probability of being observed depended only on the (continuous) outcome. Error bars are 1.96 × the Monte Carlo error.

Figure 2 and Supplementary Table S1 show the mean percent bias in the log OR when the probability of being observed depended only on the continuous outcome, with the OR for a 1 SD increase in continuous outcome of 0.75. Error bars on the figure are 1.96 times the Monte Carlo error; the Monte Carlo error quantifies uncertainty due to carrying out a finite number of simulations [10]. In this situation, imputing the binary outcome gave estimates that were similar to the complete case estimates, except at high levels of missing data, where an auxiliary variable with greater sensitivity gave estimates that were slightly less biased. Multiple imputation resulted in increased precision, particularly when the auxiliary variable had higher sensitivity.

**Fig. 3:**
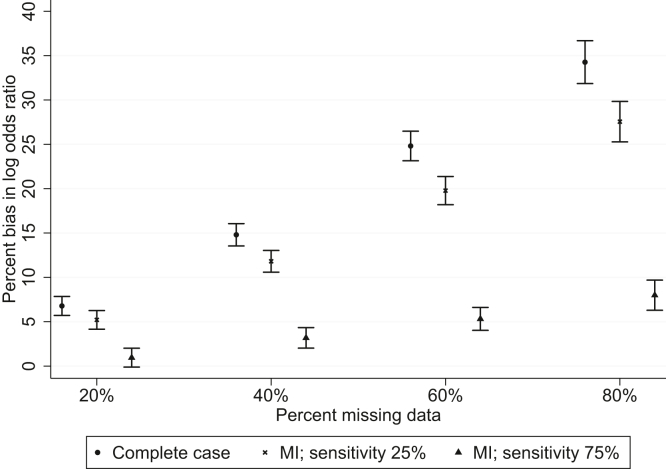
Mean percent bias in log OR when the log probability of being observed depended linearly on exposure, outcome, and their interaction. Error bars are 1.96 × the Monte Carlo error.

When the (log) probability of being observed depended on the continuous outcome, the exposure and their interaction (simulated by including an interaction term in the logistic model for the probability of being a complete case), the estimated bias in the complete case analysis ranged from 7% when 20% of the outcome data were missing to 34% when 80% were missing. Multiple imputation resulted in small reductions in bias (compared to the complete case analysis) when the auxiliary variable had low sensitivity but greater reductions when it had a higher sensitivity; precision was also increased (Fig. 3 and Supplementary Table S2). Similarly, for a given percentage of missing data, the fraction of missing information was lowest when the auxiliary variable had high sensitivity (Supplementary Table S2). When missingness was introduced in the auxiliary variable, the reductions in bias and gains in precision were lower than those seen in the equivalent scenarios in which it was fully observed (Supplementary Table S2).

### 3.3 Analysis of ALSPAC data

There were 14,684 enrolled participants alive at 1 year who had not subsequently withdrawn from the study. Of these, ALSPAC had no National Health Service number for 23 individuals and 95 explicitly dissented to linkage to their health records. This analysis is based on the remaining 14,566. Of these, 11,227 (77%) had data on smoking in pregnancy, 4,537 (31%) had depression data, and 2,718 (19%) were complete cases (individuals with data on maternal smoking in pregnancy, depression, and covariates, but not necessarily linked GP data). In addition, among the 14,566 individuals, 10,560 (72%) had sufficient GP data to generate at least one depression measure. Further details of the available data are given in Table 2. Complete cases were more likely to be female, have a mother who was nulliparous, older, and who did not smoke during pregnancy; higher socio-economic position (measured by maternal education and other factors) was also associated with being a complete case (Supplementary Table S3). In contrast, characteristics of those with GP data were similar to those among all individuals (Supplementary Table S3).

**Table 2:** Completeness of ALSPAC data by availability of GP data

Complete data on:			Linked GP data		Total
Covariates	Maternal smoking status in pregnancy	Depression status (CIS-R)	Yes^a^	No	
Yes	Yes	Yes	2,201	517	2,718
		No	2,923	1,386	4,309
	No	Yes	180	40	220
		No	280	135	415
No	Yes	Yes	830	185	1,015
		No	2,196	989	3,185
	No	Yes	478	106	584
		No	1,472	648	2,120
			10,560	4,006	14,566

*Abbreviations:* ALSPAC, avon longitudinal study of parents and children; CIS-R, revised clinical interview schedule; GP, general practitioner.

a Information on at least one of: historical, current or future diagnosis or treatment or symptoms of depression.

#### 3.3.1 Association between ALSPAC-measured and GP-recorded depression

Table 2 shows the relationship between the three GP depression outcomes and CIS-R-defined depression. Most individuals (98%) without depression according to the CIS-R did not have a current GP record for depression. However, only just over a quarter of individuals with CIS-R-measured depression had a current GP depression record (Table 3). The results were similar for historical depression. Future depression had a higher sensitivity but lower specificity.

**Table 3:** ALSPAC depression according to GP measures of depression

GP measure^a^	Present?	CIS-R diagnosis of depression	
		No	Yes
Current diagnosis or symptoms or treatment	No	3,012 (97.7%)	199
	Yes	72	71 (26.3%)
Future diagnosis or symptoms or treatment	No	2,500 (79.6%)	126
	Yes	640	156 (55.3%)
Historical diagnosis or symptoms or treatment	No	3,233 (96.2%)	217
	Yes	127	64 (22.8%)

*Abbreviations:* ALSPAC, avon longitudinal study of parents and children; CIS-R, revised clinical interview schedule; GP, general practitioner.

a The denominators vary because the numbers with historical, current, and future data on depression are different.

After mutual adjustment (for the other GP depression variables), the GP measures were all strongly associated with CIS-R-defined depression: OR = 5.04; 95% CI (3.11, 8.17); 3.14 (2.37, 4.17); and 2.31 (1.44, 3.69), for current, future, and historical depression, respectively, indicating that these would all be potentially useful auxiliary variables to include in the multiple imputation models.

#### 3.3.2 Predictors of observed ALSPAC-measured depression data

Supplementary Table S4 shows the association between covariates and the odds that CIS-R depression was observed. Since the majority of missing data was in the outcome (depression), these factors were the same as those associated with being a complete case. Table 4 shows the associations between the GP measures of depression and the odds that CIS-R depression was observed among those with complete data on maternal smoking in pregnancy, covariates, and GP data (*n* = 4,468). Using logistic regression and after adjusting for covariates (including the exposure, maternal smoking in pregnancy), individuals with a future depression record were less likely to have CIS-R depression data; the association was weaker with current and historical depression. This suggests that the outcome, depression, is likely to be MNAR conditional on the exposure and covariates; the addition of the auxiliary variables (GP-recorded depression) should reduce this dependency of missingness on the outcome–that is, should give a better approximation to missing at random (MAR).

**Table 4:** Association between GP-recorded depression and missingness in ALSPAC depression

Variable	Present?	Odds ratio (OR) (95% CI)^a^	*P*-value
Historical diagnosis or symptoms or treatment	Yes	0.88 (0.68, 1.15)	*P* = 0.4
Current diagnosis or symptoms or treatment	Yes	0.81 (0.59, 1.11)	*P* = 0.2
Future diagnosis or symptoms or treatment	Yes	0.76 (0.66, 0.88)	*P* < 0.001

*Abbreviations:* ALSPAC, avon longitudinal study of parents and children; GP, general practitioner.

a Adjusted for all covariates, including smoking in pregnancy (the exposure).

There was no evidence for interaction between maternal smoking in pregnancy and current GP-recorded depression with respect to having observed data on CIS-R-measured depression [RR for interaction between maternal smoking in pregnancy and current depression = 0.99 (0.69, 1.42), *P* = 0.9; and RR for interaction with future depression = 0.93 (0.77, 1.12), *P* = 0.5, when added to a binomial regression model including a restricted set of covariates (sex, mother's education, mother's age, parity, social class, and number of rooms)]. These covariates were selected on the basis of their strength of association with having observed depression data; only a restricted set of covariates could be included because models including additional covariates did not converge. Based on this, we would expect the estimate of the OR from the complete case logistic regression to be approximately unbiased if this association with the chance of having observed data depended on this binary measure of depression and not an underlying continuous measure of depression. However, we note that–firstly–this was not the CIS-R measure of depression but proxy is, GP-recorded depression, and–secondly–the confidence intervals (CI) for these interactions are quite wide. In the multiply imputed data, a binomial regression (with a log link) for having observed CIS-R depression showed no evidence for an interaction between maternal smoking in pregnancy and CIS-R depression (RR for interaction = 0.97 (0.65, 1.44), *P* = 0.9). Thus, under an assumption that CIS-R depression is MAR given the covariates and the GP depression variables, there would be - again - no evidence to reject the assumption required for unbiasedness of the complete case OR estimate (as above, if this association with the chance of having observed data depended on this binary measure of depression and not an underlying continuous measure).

#### 3.3.3 Relationship between maternal smoking in pregnancy and offspring depression

Table 5 gives the ORs for depression comparing offspring of mothers who smoked during pregnancy to offspring of nonsmokers obtained using the complete case analysis and MI. The complete case estimates were closer to the null than those obtained using MI; MI resulted in increased precision.

**Table 5:** Relationship between smoking in pregnancy and offspring depression: OR estimates obtained from complete case and MI analyses

Analysis approach	Crude OR (95% CI)	Adjusted^a^ OR (95% CI)	Gain in precision^b^ (adjusted log OR)
Complete case (*n* = 2,718)	1.72 (1.20, 2.46)	1.36 (0.92, 2.02)	n/a
MI^c^ (*n* = 14,566)	1.86 (1.44, 2.40)	1.46 (1.06, 2.01)	24%

*Abbreviations:* MI, multiple imputation; OR, odds ratio.

a Adjusted for sex, mothers age, parity, & education, family occupational social class, maternal and paternal antenatal depression and anxiety, housing tenure, and number of rooms in house.

b Standard error (log OR) from complete case analysis/standard error (log OR) from MI, expressed as a percentage decrease/increase.

c Including linked GP data and mother ever smoked (from ALSPAC) as auxiliary variables.

## 4 Discussion

The robustness of logistic regression to missing data is not maintained when the outcome is a binary version of an underlying continuous measure. Our results show that, where a binary outcome has been derived from an underlying continuous measure, when the probability of a being a complete case depends independently on the exposure and the underlying continuous outcome (or only on the continuous outcome), then estimates of the OR for exposure from a complete case analysis will generally be subject to bias. This bias will be relatively small if the association between the outcome and the probability of being observed is weak but could be large if this association is strong. If the association between the continuous outcome and the probability of being a complete case varies by levels/categories of the exposure, the bias could be substantial. When the probability of being a complete case is independently associated with the exposure and outcome (or only with the outcome), including a misclassified form of the missing binary outcome as an auxiliary variable in MI will result in similar estimates of the log OR compared to the complete case analysis for low to moderate amounts of missing data, but the estimates will be less biased if the amount of missing data is high and the proxy has high sensitivity (and specificity; note that because we did not vary the specificity in our simulations, we cannot tell whether the same would hold if the specificity were lower). MI will also lead to increases in precision, particularly when the proxy has high sensitivity (and specificity). If an interaction is present (nonindependent association between exposure and outcome on the probability of having complete data), imputing the binary outcome will lead to relatively large bias reductions if the proxy has high sensitivity (and specificity); otherwise, the bias reductions are likely to be small. Since a standard implementation of MI assumes the data are MAR, the reductions in bias in MI relative to the complete case analysis result from getting closer to MAR.

In the ALSPAC example, we used three (rather than one) auxiliary variables with sensitivities 22% (historical), 26% (current), and 55% (future depression) and specificities 96%, 98%, and 80%, respectively, in relation to the missing binary outcome. These were independently associated with ALSPAC-measured depression so would be better than a single auxiliary variable with sensitivity 25% but may not predict ALSPAC-measured depression as accurately as a single variable with sensitivity 75%. Although there was no evidence for an interaction between the exposure and outcome with respect to the probability of having complete data, this analysis used the proxy outcome measure, GP-recorded depression, rather than the CIS-R measure of depression. However, in the imputed data, there was no interaction between the exposure and the CIS-R measure of depression in a log link model for the probability of being a complete case. A key difference in the ALSPAC analysis, however, was the fact that most of the covariates–including the exposure–were also partially observed, with some also potentially MNAR. Thus, since the MI estimate of the OR in this example was slightly higher than the complete case estimate, the simulations suggest that the MI estimate could be subject to a small amount of (upward) bias due to a violation of the MAR assumption. The complete case analysis could also be upwardly biased if the probability of being a complete case depended on an underlying continuous measure of depression; it is not possible to determine this from the observed data but - given that the association with (binary) GP-recorded depression was not strong - any such bias is also likely to be small. Of course, there are likely to be other sources of bias in the estimate - most notably, residual confounding [11]. Similarly, and as discussed by Bartlett et al., the estimate from both the complete case analysis and MI would also be biased if the outcome model were incorrectly specified [2].

In terms of using auxiliary data, our findings are in line with previous research showing that the inclusion of auxiliary variables in MI can increase precision and reduce bias as long as the correlation between the auxiliary variable(s) and the variable with missing data is reasonably high [[12], [13], [14]].

Our study has several limitations. In particular, the simulations did not match the data example exactly. Firstly, the ALSPAC example also included covariates, many of which were subject to missing data themselves and predictors of being a complete case, whereas covariates were not included in the simulations. Secondly, in ALSPAC, GP data were not available for all participants. Although the distribution of most characteristics was similar among the subgroup with GP data compared to among the overall sample, individuals living in owned or mortgaged accommodation were more likely than those in rented accommodation to have linked GP data (Supplementary Table S3).

In summary, it is already known that when a continuous outcome is MNAR, a complete case analysis will result in a biased estimate of the exposure-outcome association. Our results suggest that if this outcome is dichotomized or if the underlying continuous outcome is not measured (such that only a binary form is available) and a complete case logistic regression is used, this is also likely to produce a biased estimate of the exposure OR. This bias is likely to be small if the probability of being a complete case is independently associated with the exposure and continuous outcome (or only associated with the continuous outcome) and the association between the outcome and the probability of being observed is not strong. As with logistic regression where missingness depends directly on the binary outcome, this bias could be large if there is an interaction between the exposure and continuous outcome with respect to the probability of being a complete case. This bias could be reduced by including one or more proxies for the missing outcome as auxiliary variables in MI. If such proxies are available, we would recommend using MI over a complete case analysis because, in practice, it would be difficult to rule out an interaction. Note that it would also be important to carry out sensitivity analyses to explore the robustness of the findings to any assumptions made about the missing data mechanism.
