# Supplementary material for: Complete case logistic regression with a dichotomised continuous outcome led to biased estimates
Source: J Clin Epidemiol. 2023 Feb;154:33–41. doi: 10.1016/j.jclinepi.2022.11.022 (PMC10322727; doi:10.1016/j.jclinepi.2022.11.022)
Supplement: Supplementary Material [file mmc1.docx]

Supplementary material

1. **Technical details and formulae**
   1. **Condition required for a complete case logistic regression to produce an (asymptotically) unbiased estimate of the exposure odds ratio**

If R is the response (observation) indicator (such that R=1 for complete cases and R=0 otherwise) and Y, X, and C are the outcome, exposure and confounders, respectively, the complete case exposure odds ratio is asymptotically unbiased provided P(R=1|Y,X,C) = f(X,C)⨯g(Y,C) for some functions f(X,C) and g(Y,C).

- 1. **General expression for the odds ratio consistently estimated by a complete case logistic regression**

Here we let X denote the exposure, Y_cts_ the continuous outcome, and Y_bin_ the binary outcome. As before, let R denote the observation indicator (R=1 if observed, R=0 if not). We assume that R depends only on X and Y_cts_ and, given these, not on Y_bin_, with

$$P\left( R=1 | X,Y_{cts} \right)=\psi(X,Y_{cts})$$

for some function $\psi(X,Y_{cts})$.

The complete case analysis (CCA) consistently estimates the odds ratio among those with R=1. To derive the odds ratio that a CCA consistently estimates, we find an expression for P(Y_bin_=1 | X=x, R=1):

| $P\left( Y_{bin}=1 \vert X=x,R=1 \right)=\int P\left( Y_{bin}=1,Y_{cts} \right\vert X=x,R=1) dY_{cts}$ $=\int P\left( Y_{bin}=1 \vert Y_{cts},X=x,R=1 \right)f\left( Y_{cts} \vert X=x,R=1 \right)dY_{cts}$ $= \int P\left( Y_{bin}=1 \vert Y_{cts} \right)f\left( Y_{cts} \vert X=x,R=1 \right)dY_{cts}$ | (1) |
| --- | --- |

We now expand the second term in the integral as

| $f\left( Y_{cts} \vert X=x,R=1 \right)=\frac{f\left( Y_{cts},X=x,R=1 \right)}{P\left( X=x,R=1 \right)}$ $=\frac{P\left( R=1 \vert Y_{cts},X=x \right)f\left( Y_{cts} \vert X=x \right)P\left( X=x \right)}{P\left( R=1 \vert X=x \right)P\left( X=x \right)}$ $=\frac{\psi(x,Y_{cts})f\left( Y_{cts} \vert X=x \right)}{\int\psi\left( x,y_{cts} \right)f\left( y_{cts} \vert X=x \right)dy_{cts}}$ | (2) |
| --- | --- |

Substituting (2) in to (1), we have

| $P\left( Y_{bin}=1 \vert X=x,R=1 \right)= \int P\left( Y_{bin}=1 \vert Y_{cts} \right)f\left( Y_{cts} \vert X=x,R=1 \right)dY_{cts}$ $=\frac{\int P\left( Y_{bin}=1 \vert Y_{cts} \right)\psi(x,Y_{cts})f\left( Y_{cts} \vert X=x \right)dY_{cts}}{\int\psi\left( x,Y_{cts} \right)f\left( Y_{cts} \vert X=x \right)dY_{cts}}$ | (3) |
| --- | --- |

From this quantity, given specifications for the relationship between the binary outcome and continuous outcome ($P\left( Y_{bin}=1 | Y_{cts} \right))$, for example a logistic model, the distribution of the continuous outcome conditional on the exposure ($f\left( Y_{cts} | X=x \right)$), for example normal, and the missingness/observation function $\psi(x,Y_{cts})$, the odds of Y_bin_=1 in the complete cases among those with X=x can be obtained for a given value of x. From this the odds ratio comparing X=1 to X=0 can be calculated. We note that the expression given in (3) above, and hence also the CCA odds ratio, does not depend on the marginal distribution of the exposure (i.e. the prevalence if X is binary).

To calculate the full population odds ratio the preceding expression can be used, setting

$$P\left( R=1 | X,Y_{cts} \right)=\psi\left( X,Y_{cts} \right)=1$$

which gives

| $P\left( Y_{bin}=1 \vert X=x \right)=\int P\left( Y_{bin}=1 \vert Y_{cts} \right)f\left( Y_{cts} \vert X=x \right)dY_{cts}$ | (4) |
| --- | --- |

**Probability of being a complete case depending independently on exposure and continuous outcome**

Now consider the special case where the probability of being observed depends independently on X and Y_cts_ such that

$$P\left( R=1 | X,Y_{cts} \right)=\psi\left( X,Y_{cts} \right)$$

$$=p_{1}(X)p_{2}(Y_{cts})$$

for some functions $p_{1}\left( X \right)$ and $p_{2}(Ycts)$.

Then substituting into (3) we obtain

| $P\left( Y_{bin}=1 \vert X=x,R=1 \right)=\frac{\int P\left( Y_{bin}=1 \vert Y_{cts} \right)p_{1}\left( x \right) p_{2}\left( Y_{cts} \right)f\left( Y_{cts} \vert X=x \right)dY_{cts}}{\int p_{1}\left( x \right) p_{2}\left( Y_{cts} \right)f\left( Y_{cts} \vert X=x \right)dY_{cts}}$ $=\frac{\int P\left( Y_{bin}=1 \vert Y_{cts} \right) p_{2}\left( Y_{cts} \right)f\left( Y_{cts} \vert X=x \right)dY_{cts}}{\int p_{2}\left( Y_{cts} \right)f\left( Y_{cts} \vert X=x \right)dY_{cts}}$ | (5) |
| --- | --- |

Note the $p_{1}\left( x \right)$ term has cancelled, implying that the CCA OR for the exposure effect does not depend on the form of $p_{1}\left( X \right).$

- 1. **Formulae used to calculate the bias in the complete case log odds ratio using the above expressions and in the simulation study**

The following were substituted into the equations in 1.2 above to calculate the bias in the CCA log odds ratio; they were also used to generate the simulated datasets.

1. $f\left( Y_{cts} | X=x \right)$

The continuous depression score for individual $i$was generated conditional on smoking such that:

|  | $\text{Depression scor}\text{e}_{i}= \beta_{0}+\beta_{1}\times\left( \text{smoke\_pre}\text{g}_{i} \right)+ \varepsilon_{i}$ | (6) |
| --- | --- | --- |

where *smok_preg* is maternal smoking in pregnancy, coded 0/1, and $\varepsilon$ is error, following a normal distribution with mean 0 and variance $\sigma^{2},$calculated to give the score a variance of 1 marginally.

1. $P\left( Y_{bin}=1 | Y_{cts} \right)$

The binary depression measure was assumed to depend on the depression score via logistic regression (Equation 7):

| logit($\text{p\_dep}\text{s}_{\text{i}})=\alpha_{0}+ \alpha_{1} \times(d\text{epression scor}\text{e}_{i})$ | (7) |
| --- | --- |

where p_deps_i_ represents the probability that an individual was classified as having depression (in the study data) and with $\alpha_{0}$= -6.9875 and $\alpha_{1}=$ 6.5, chosen using trial and error to give a prevalence of 15% and such that this logistic function was very steep (Supplementary Figure S1) – i.e. generating a strong relationship between the depression score and the binary depression measure.

The analysis model is given by Equation 8.

|  | $\text{logit(}\text{p\_dep}\text{s}_{\text{i}}\text{)}= \mu_{0}+\mu_{1}\times\left( \text{smoke\_pre}\text{g}_{\text{i}} \right)$ | (8) |
| --- | --- | --- |

The regression coefficient $\beta_{1}$ for maternal smoking in pregnancy from Equation 6 was set at 0.2317; this was chosen by trial and error to give a log OR for depression of 0.405 (to 3 decimal places) which gave an OR of 1.50 comparing those whose mother smoked to those whose mother did not smoke during pregnancy). The prevalence of exposure (maternal smoking) was set at 25%; thus, $\beta_{0}\text{ }\text{and}\text{ σ}$ in Equation 6 were given by:

$\beta_{0}=0-(0.2317\times0.25)$ to give the depression score a mean of 0 and

$\sigma=\sqrt{1-({0.2317)}^{2}\times0.25\times0.75)}$ to give it a variance of 1.

(For simulations only): The linked (binary) GP measure of depression was created – using a logistic function – to give different sensitivities in relation to the study’s binary measure (Equation 9).

|  | $\text{p\_GPde}\text{p}_{\text{i}}=\frac{1}{1+\exp(\rho\times(\text{depression scor}e_{i}-\theta))}$ | (9) |
| --- | --- | --- |

Values of $\rho\text{and} \theta$ were chosen (using trial and error) to give sensitivities of 25% and 75% and a specificity of 97.5%.

**Probability of being a complete case dependent independently on exposure and continuous outcome**

1. $p_{1}\left( X \right) \text{and }p_{2}(Y_{cts})$

As noted in 1.2, the expression for the complete case estimate of the exposure odds ratio does not depend on the form of $p_{1}\left( X \right)$. Thus, we focused only on $p_{2}{(Y}_{cts})$: this probability (of the outcome being observed) was assumed to depend on the continuous outcome via logistic regression (Equation 10).

|  | $\text{logit}\text{(}p_{2i})= \gamma_{0}+\gamma_{1}\times\text{depression score}_{i}$ | (10) |
| --- | --- | --- |

To show how the bias varied as the strength of association between the continuous outcome and the probability of the outcome being observed and the percentage of missing data varied, we calculated the values of $\gamma_{0}$ that gave a given percentage of missing data for different values of $\gamma_{1}$ [for $\gamma_{1}$= ln(0.90), ln(0.75), ln(0.50) and ln(0.25)]. In the simulations, $\gamma_{1}$ was fixed as ln(0.75).

**Simulations only: probability of being observed dependent multiplicatively on the exposure, continuous outcome and their interaction**

The probability of being observed were generated from the logistic model shown in Equation 11, so that the logarithm of the probability of the outcome being observed (being a complete case) depended on exposure, outcome and their interaction. The values of $\tau_{0}$ were chosen using trial and error to produce given percentages of missing data. In these scenarios with an interaction, $\tau_{1}$, $\tau_{2},$and $\tau_{3}$ were fixed at ln(0.7), ln(0.9), and ln(1.1), respectively. Note that this interaction on the logit scale implies a multiplicative interaction between the exposure and outcome with respect to the probability of being observed, such that a complete case analysis is not expected to be (asymptotically) unbiased.

|  | $\text{logit(P(observed})_{i})= \tau_{0}+ \tau_{1}\times\text{smoke\_preg}_{i} {+ \tau}_{2}\times\text{depression score}_{i}+ \tau_{3}\times\text{depression score}_{i}\times\text{smoke\_preg}_{i}$ | (11) |
| --- | --- | --- |

1. **Linkage to GP data**

As part of the Secure Anonymised Information Linkage (SAIL) project [1], the NHS Wales Information Service (NWIS) and the Health Informatics Research Unit (HIRU) at the University of Swansea have established a method through which individual level data from multiple sources can be linked and analysed in a secure setting, including data from primary care electronic patient records. ALSPAC, working with the SAIL team, developed two methods to extract GP records which took advantage of the SAIL infrastructure:

Pilot extraction: In 2012 ALSPAC carried out a pilot extraction which included only individuals who had provided explicit consent. The methods for this extraction have been described in a previous paper [2].

Main extraction: The NHS South West Commissioning Support Unit (SWCSU) has developed a governance framework and data extraction mechanism which secured opt-in assent from GP practices for the extraction of records and their use for SWCSU approved purposes. Invitations to participate in this system were made to all practices in the Bristol, North Somerset, Somerset and South Gloucestershire (BNSSSG) clinical commissioning group. The extraction mechanism is provided by EMIS, which supplies software systems to the majority of practices in the BNSSSG area. ALSPAC gained approval from the SWCSU Security and Informatics Group to extract participants’ GP records. SWCSU informed all participating practices about this agreement and gave them opportunity to opt-out.

For both the pilot study and the main extraction, the methods after extraction were identical. The extracted records were pseudonymised and securely transferred into the infrastructure at Swansea University using SAIL’s “split file” method and adhered to NHS standards of encryption and security, as described previously [2].

References

1. Ford, D.V., et al., *The SAIL Databank: building a national architecture for e-health research and evaluation.* BMC Health Serv Res, 2009. **9**: p. 157.

2. Cornish, R.P., et al., *Defining adolescent common mental disorders using electronic primary care data: a comparison with outcomes measured using the CIS-R.* BMJ Open, 2016. **6**(12).

1. **Additional figures and tables**

Supplementary Figure S1: Simulated relationship between the continuous depression score and the probability of depression (p_deps) being equal to 1 for a prevalence of 15%.


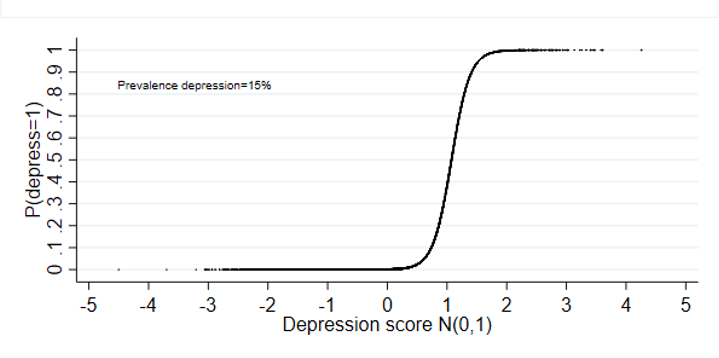


Supplementary Figure S2: Percent bias in log odds ratio from a complete case analysis for a full population exposure log odds ratio of 0.405 when the probability of being observed only depends on the continuous outcome and this probability increases as the continuous outcome increases


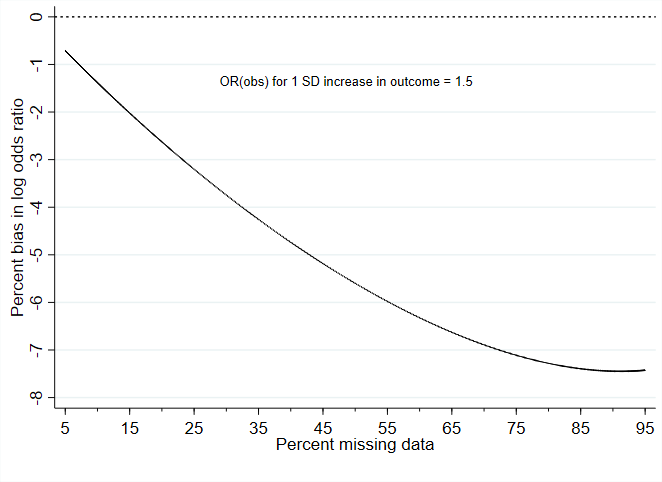


Supplementary Table S1: Simulation results - complete case and MI estimates of the log odds ratio (full population log odds ratio = 0.405) when the probability of being observed depended only on the continuous outcome

| Factor 1:  % missing | Complete case | | Factor 2:  sensitivity | MI | | | |
| --- | --- | --- | --- | --- | --- | --- | --- |
|  | Mean estimate (empirical SE) | % bias (mcse) |  | Mean estimate (empirical SE) | % bias  (mcse) | Gain in precision | FMI |
| 20% | 0.409 (0.070) | 0.9% (0.5%) | 25 | 0.410 (0.069) | 1.2% (0.5%) | 2% | 19% |
|  |  |  | 75 | 0.409 (0.064) | 1.0% (0.5%) | 8% | 11% |
| 40% | 0.414 (0.084) | 2.2% (0.7%) | 25 | 0.415 (0.080) | 2.4% (0.6%) | 5% | 38% |
|  |  |  | 75 | 0.414 (0.070) | 2.1% (0.6%) | 21% | 23% |
| 60% | 0.416 (0.110) | 2.8% (0.8%) | 25 | 0.420 (0.102) | 3.8% (0.8%) | 8% | 58% |
|  |  |  | 75 | 0.414 (0.081) | 2.1% (0.8%) | 37% | 41% |
| 80% | 0.422 (0.162) | 4.2% (1.3%) | 25 | 0.422 (0.148) | 4.2% (1.2%) | 8% | 78% |
|  |  |  | 75 | 0.412 (0.109) | 1.7% (0.9%) | 50% | 65% |

Supplementary Table S2: Simulation results - complete case and MI estimates of the log odds ratio (full population log odds ratio = 0.405) when the probability of being observed depended multiplicatively on exposure, continuous outcome and their interaction

| Factor 1:  % missing | Complete case | | Factor 2:  sensitivity | Factor 4: 25% missing in linked variable | MI | | | |
| --- | --- | --- | --- | --- | --- | --- | --- | --- |
|  | Mean estimate (empirical SE) | % bias (mcse) |  |  | Mean estimate (empirical SE) | % bias  (mcse) | Gain in precision | FMI |
| 20% | 0.432 (0.072) | 7% (0.5%) | 25 | No | 0.426 (0.070) | 5% (0.5%) | 3% | 20% |
|  |  |  | 75  75 | No  Yes | 0.409 (0.068)  0.417 (0.070) | 1% (0.5%)  3% (0.5%) | 6%  4% | 11%  14% |
| 40% | 0.465 (0.082) | 15% (0.6%) | 25 | No | 0.453 (0.078) | 12% (0.6%) | 5% | 41% |
|  |  |  | 75  75 | No  Yes | 0.418 (0.074)  0.432 (0.077) | 3% (0.6%)  7% (0.6%) | 11%  7% | 25%  30% |
| 60% | 0.505 (0.107) | 25% (0.8%) | 25 | No | 0.485 (0.102) | 20% (0.8%) | 5% | 62% |
|  |  |  | 75  75 | No  Yes | 0.427 (0.083)  0.443 (0.088) | 5% (0.7%)  9% (0.7%) | 29%  21% | 43%  48% |
| 80% | 0.544 (0.155) | 34% (1.2%) | 25 | No | 0.517 (0.146) | 28% (1.1%) | 6% | 81% |
|  |  |  | 75  75 | No  Yes | 0.437 (0.109)  0.453 (0.116) | 8% (0.9%)  12% (0.8%) | 42%  34% | 67%  71% |

Supplementary Table S3: Characteristics of the ALSPAC-enrolled sample and complete cases

| Characteristic |  | Enrolled singletons and twins, alive at one year, not subsequently withdrawn (n=14,566)^1^ | Complete cases (n=2,718) | Those with GP data needed to measure depression (n=10,560) |
| --- | --- | --- | --- | --- |
| Sex | Male  Female | 7,645 (51%)  7,902 (49%) | 802 (43%)  1,067 (57%) | 5,297 (50%)  5,263 (50%) |
| Maternal age | <20  20-24  25-29  30-34  35+ | 647 (5%)  2,679 (19%)  5,358 (39%)  3,809 (27%)  1,371 (10%) | 29 (1%)  312 (11%)  1,038 (38%)  979 (36%)  360 (13%) | 495 (5%)  1,904 (19%)  3,896 (39%)  2,758 (28%)  953 (10%) |
| Parity | 0  1  2+ | 5,728 (45%)  4,491 (35%)  2,601 (20%) | 1,346 (50%)  796 (29%)  576 (21%) | 4,104 (44%)  3,272 (35%)  1,895 (20%) |
| Smoking in pregnancy | No  Yes | 7,645 (68%)  3,582 (32%) | 2,308 (85%)  410 (15%) | 5,568 (68%)  2,582 (32%) |
| Maternal education | O level/lower  A level  Degree/higher | 7,967 (65%)  2,766 (22%)  1,579 (13%) | 1,346 (50%)  796 (29%)  576 (21%) | 5,903 (66%)  1,958 (22%)  1,037 (12%) |
| Family occupational social class | Non-manual  Manual | 9,184 (81%)  2,222 (19%) | 2,433 (90%)  285 (10%) | 6,581 (80%)  1,679 (20%) |
| Housing tenure | Mortgaged/owned  Private rented  Other | 9,473 (73%)  921 (7%)  2,523 (20%) | 2,413 (89%)  92 (3%)  213 (8%) | 6,952 (74%)  555 (6%)  1,828 (20%) |
| Number of rooms in home | Median (IQR) | 5 (4-6) [n=12,786] | 5 (4-6) | 5 (4-6) [n=9,244] |
| Maternal depression score (EPDS) | Median (IQR) | 6 (3-10) [n=11,875] | 6 (3-9) | 6 (3-10) [n=8,631] |
| Paternal depression score (EPDS) | Median (IQR) | 3 (1-6) [n=9,614] | 3 (1-6) | 3 (1-6) [n=6,957] |
| Maternal anxiety score | Median (IQR) | 4 (2-7) [n=11,945] | 4 (2-6) | 4 (2-7) [n=8,679] |
| Paternal anxiety score | Median (IQR) | 2 (1-4) [n=9,564] | 2 (1-4) | 2 (1-5) [n=6,909] |

1. Denominators vary because the variables come from different questionnaires and have different completion rates.

Supplementary Table S4: Predictors of the odds of observing ALSPAC-measured depression: covariates (n= 7,027 with complete covariate data)

| Factor | Level | OR (95% CI)^1^ | p-value |
| --- | --- | --- | --- |
| Smoking in pregnancy  Sex  Mother’s education  Mother’s age at birth  Parity  Maternal depression  Maternal anxiety  Paternal depression  Paternal anxiety  Housing tenure  Number of rooms  Family occupational social class | Yes vs no  Female vs male  O level/lower  A level  Degree  <20  20-24  25-29  30-34  35+  0  1  2+  Per 1 point increase  Per 1 point increase  Per 1 point increase  Per 1 point increase  Mortgaged /owned  Private rented  Council/HA/other  Per 1 room increase  Manual vs non-manual | 0.64 (0.56, 0.73)  1.59 (1.45, 1.75)  1.00  1.43 (1.27, 1.61)  1.59 (1.35, 1.85)  1.00  1.82 (1.19, 2.86)  1.96 (1.28, 3.03)  2.56 (1.64, 3.85)  2.78 (1.79, 4.35)  1.00  0.78 (0.69, 0.88)  0.61 (0.52, 0.71)  1.00 (0.99, 1.02)  0.99 (0.97, 1.01)  0.99 (0.97, 1.01)  1.02 (1.00, 1.04)  1.00  0.51 (0.39, 0.65)  0.75 (0.62, 0.90)  1.09 (1.04, 1.14)  0.80 (0.68, 0.93) | p<0.001  p<0.001  p<0.001  p<0.001  p<0.001  p=0.7  p=0.3  p=0.4  p=0.1  p<0.001  p<0.001  p=0.005 |
